# Serum Vitamin D Levels and Risk of Iron Deficiency Anemia in Adults: A Cross‐Sectional Study and Mendelian Randomization Analysis

**DOI:** 10.1002/fsn3.4746

**Published:** 2025-02-24

**Authors:** Leifei Chen, Nanyuan Gu, Kai Qiu, Hui Chen, Fu Tian, Yang Chen, Longhuan Zeng

**Affiliations:** ^1^ Department of High Dependency Unit Hangzhou Geriatric Hospital Hangzhou China

**Keywords:** cross‐sectional study, iron deficiency anemia, Mendelian randomization, NHANES, vitamin D

## Abstract

This study focuses on the potential role of vitamin D in the diagnosis and treatment of iron deficiency anemia (IDA), and evaluates causation using Mendelian randomization (MR). This cross‐sectional study collected clinical information from 3728 respondents in the National Health and Nutrition Examination Survey (NHANES) cycles of 2005–2010 and 2015–2018. To determine the link between serum vitamin D levels and the risk of IDA, we established a robust nomogram. Calibration and net clinical benefits were examined through calibration curves and decision curve analysis (DCA). Restricted cubic spline (RCS) models were utilized to explore the relationship between the two. In addition, the data of single nucleotide polymorphism (SNP) related to vitamin D and IDA were obtained from open biological databases. The main analytical method for the MR analysis was the inverse variance weighted (IVW) method. A series of sensitivity analyses were conducted to evaluate pleiotropy. Our cross‐sectional study showed that, after extensive adjustments, serum vitamin D levels remained an independent risk factor for predicting the development of IDA. The risk of developing IDA was significantly lower for participants in the highest quartile subgroup with vitamin D levels ≥ 78.1 nmol/L compared to those in the lowest quartile with vitamin D levels ≤ 42.8 nmol/L (*p* < 0.001). In terms of gender, serum vitamin D primarily exhibited a protective effect against IDA in females (OR:0.98, 95% CI: 0.98–0.99, *p* < 0.001). A non‐linear relationship between the two was found (*p* < 0.001 for non‐linear relationship). Meanwhile, using the IVW method in MR analysis, we identified a bidirectional causal relationship. The results of our cross‐sectional analysis demonstrated a negative correlation between serum vitamin D levels and IDA. Additionally, genetic evidence from the MR analysis supported an association between serum vitamin D levels and IDA.

## Graphical abstract

The results of our cross‐sectional analysis demonstrated a negative correlation between serum vitamin D levels and IDA. A non‐linear relationship between the two was found.

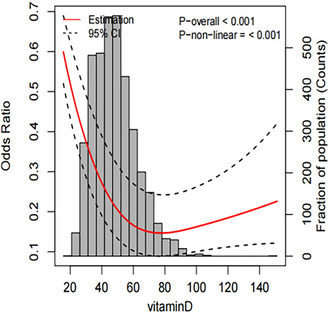

## 1 Introduction

Iron deficiency anemia (IDA) is a pervasive and critical global health concern that affects various organ functions, including cognitive development, immune mechanisms, and physical capacity (McCann, Perapoch Amadó, and Moore 2020). Globally, 30%–50% of individuals lack adequate vitamin D, which plays a pivotal role in bone metabolism, immune defense, and potentially the prevention of non‐communicable diseases like cardiovascular ailments and diabetes. Vitamin D is primarily obtained through sunlight exposure on the skin, with a smaller portion obtained through diet. Due to its long half‐life, relative stability, and high plasma concentration, its content is usually assessed clinically by measuring its converted active substance, 25‐hydroxyvitamin D [25(OH)D] (Zhou, Luo, and Qin 2019; Vanherwegen, Gysemans, and Mathieu 2017). The influence of vitamin D on IDA remains controversial. Some theories propose that vitamin D may stimulate erythropoiesis by directly acting on erythrocyte precursors, thereby increasing iron stores and reducing pro‐inflammatory cytokines. Other studies suggest that vitamin D might modulate the immune system, potentially altering iron homeostasis and hepcidin expression (Smith and Tangpricha 2015; Alfhili et al. 2022; Soepnel et al. 2023; Braithwaite et al. 2019; Madar et al. 2016; Seamans and Cashman 2009).

We first conducted a cross‐sectional of data collected from the National Health and Nutrition Examination Survey (NHANES) database to assess the effect of vitamin D levels on IDA risk (Ahluwalia et al. 2016). Furthermore, in recognition of the limitations of observational studies, we complemented our analysis with the application of Mendelian randomization (MR). MR allows for the assessment of causal relationships between circulating vitamin D levels and IDA risk by leveraging genetic instrumental variables (IVs). This approach capitalizes on the random allocation of genetic variation during gamete formation, rendering it less susceptible to confounding factors and reverse causation commonly encountered in traditional observational studies (Sekula et al. 2016; Didelez and Sheehan 2007). By elucidating the relationship between vitamin D and IDA, our research aimed to provide insights into potential therapeutic interventions and public health strategies.

## 2 Materials and Methods

### 2.1 Cross‐Sectional Study

#### 2.1.1 Study Design and Participants

Data from the NHANES databases from 2005 to 2010 and 2015 to 2018 were analyzed. The NHANES is a significant initiative sanctioned by the NCHS Research Ethics Review Board. In total, 102,956 individuals participated in the surveys conducted between 2005–2010 and 2015–2018. However, 99,878 individuals under the age of 18 or lacking complete clinical data were excluded from the current study. Thus, the final sample population used in our analysis was comprised of 3728 participants with complete serum vitamin D levels and IDA data. General patient information included the patients' gender, age, history of renal insufficiency, diabetes, hypertension, race, marital status, body mass index (BMI), poverty income ratio (PIR), education level, and history of alcohol use. Laboratory test results encompassed hemoglobin (Hb), mean corpuscular volume (MCV), mean corpuscular hemoglobin (MCH), mean corpuscular hemoglobin concentration (MCHC), ferritin, transferrin receptor, cystemic immune‐inflammation index(SII), serum total folate, RBC folate, total calcium, and C reactive protein (CRP) levels. SII was derived from platelet count ×neutrophil count/lymphocyte count and presented as ×10^5^ cells/ml.

#### 2.1.2 Iron Deficiency Anemia

**FIGURE 1:**
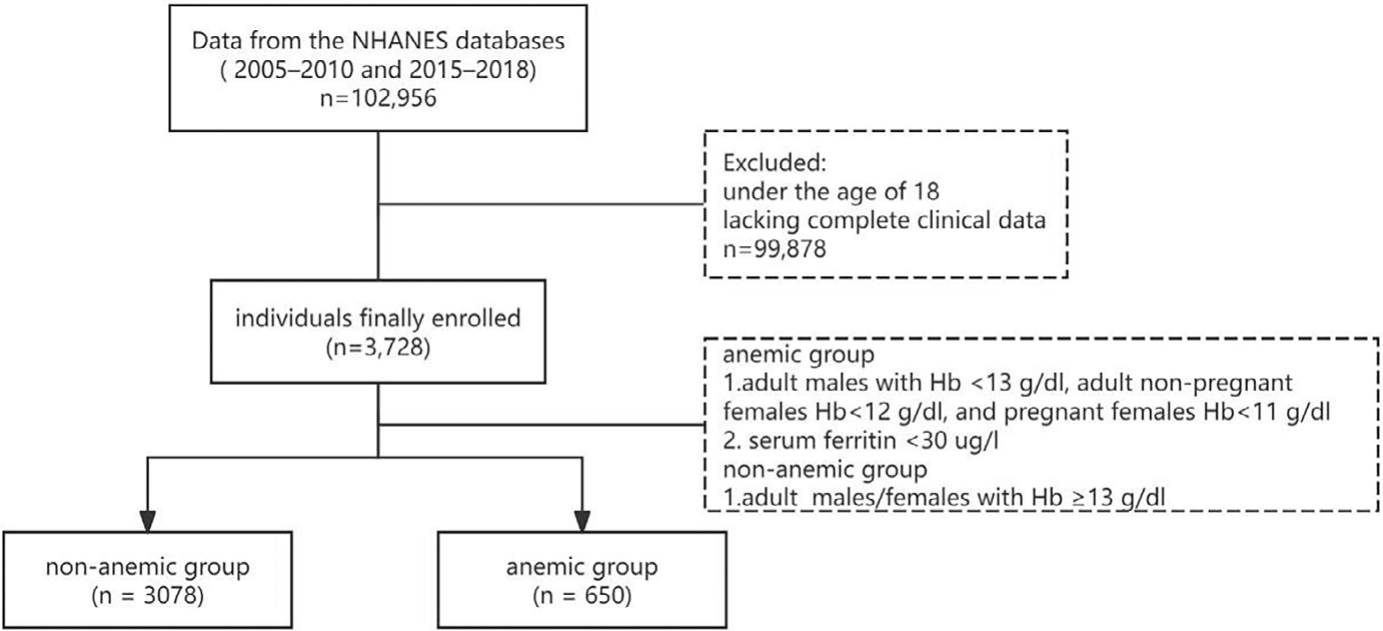
Flowchart for study subject selection.

According to the World Health Organization's definition of anemia, adult males with Hb < 13 g/dL, adult non‐pregnant females < 12 g/dL, and pregnant females < 11 g/dL are considered to be anemic (Chan 2011). China's “Multidisciplinary Expert Consensus on the Diagnosis, Treatment, and Prevention of Iron Deficiency Disorders and Iron Deficiency Anemia” defines IDA patients as anemic individuals with serum ferritin < 30 μg/L (Red Blood Cell Diseases (Anemia) Group of the Hematology Branch of the Chinese Medical Association 2022). The study design is outlined in Figure 1.

#### 2.1.3 Statistical Analysis

All statistical analyses were performed using R version 4.3.1 and SPSS version 25.0. For normally distributed data, group comparisons were carried out using two‐sample independent t‐tests and values are given as x ± s. For non‐normally distributed data, the Wilcoxon rank sum test was applied for group comparisons of paired subjects, and values are given as medians (M) with interquartile ranges (Q1, Q3). For categorical data, the chi‐squared test was used to compare groups, and results are presented as frequencies and proportions. The univariate analysis yielded variables with *p* < 0.05, which were then incorporated into the logistic regression analysis to identify independent risk factors for the development of IDA in patients. Serum vitamin D levels were categorized into four quartiles, using the first quartile (T1) as the reference.

Independent predictors of IDA were incorporated into a predictive model, visualized through a nomogram, with model stability evaluated using calibration curves and decision curve analysis (DCA). Restricted cubic spline (RCS) curves were employed to explore the non‐linear relationship between serum vitamin D levels and IDA.

We developed four logistic regression models to assess the association between iron deficiency anemia and serum vitamin D. The first model was not adjusted for its covariates. The first model was not adjusted for its relevant covariates and only roughly estimated the correlation. The second model was further adjusted for CRP, SII. The third model controlled for gender, race BMI, PIR, and education level. The fourth model adjusted for RBC folate, age, marital status, hypertension, renal insufficiency, and serum total folate. We also examined whether this association differed between men and women. The models were specified as follows:

- Model 1: crude model.
- Model 2: adjusted for CRP, SII.
- Model 3: Model 2 + gender, PIR, BMI, race, education level.
- Model 4: Model 3 + RBC folate, age, marital status, hypertension, renal insufficiency, serum total folate.

### 2.2 Mendelian Randomization Study

#### 2.2.1 Study Design and Data Sources

**FIGURE 2:**
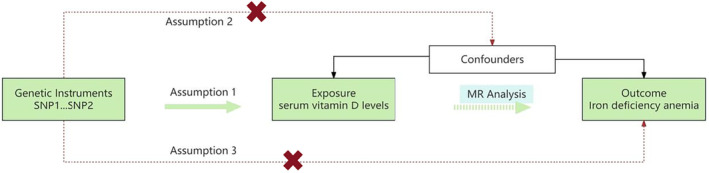
Overall design of the study.

Three core assumptions need to be met when performing MR analysis: (1) correlation assumption: there is a strong correlation between IVs and vitamin D levels; (2) independence assumption: IVs cannot be associated with confounding factors; (3) exclusivity assumption: IVs cannot directly cause IDA, but can only indirectly affect the occurrence of IDA through the sole pathway of vitamin D levels (Sekula et al. 2016). Figure 2 shows the overall design of our study, which employs MR analysis using a two‐sample approach to investigate the causal relationship between serum vitamin D levels and IDA.

The data used in this study were obtained from the IEU Open GWAS Project database and the FinnGen study, which focuses on European populations and ensures that there are no ethnic differences between the exposed and outcome populations. The genetic data on vitamin D levels were obtained from the FinnGen study, which includes populations with vitamin D deficiency. This dataset includes association analyses of 1,048,575 single nucleotide polymorphisms (SNPs). The data on IDA were obtained from an online dataset by Dönertaş et al. (GWAS ID: ebi‐a‐GCST90038659) (Dönertaş et al. 2021); this dataset provides genome‐wide analysis data for 484,598 European individuals of both sexes, and includes association analyses of 9,587,836 SNPs.

#### 2.2.2 Instrumental Variables

To ensure the independence and high correlation between exposure factors and outcome variables, we selected significant SNPs across the genome as instrumental variables, based on the whole genome information provided by the 1000 Genomes Project. The *p*‐value threshold for genome‐wide significance for coagulation factors was set at < 1 × 10^−5^, the linkage disequilibrium parameter (r^2^) was set at 0.001, and the genetic window was set at 10,000 kb. For the data on serum vitamin D levels, instrumental variables without linkage effects were screened. SNPs with an F‐value less than 10 were excluded to avoid weak instrumental variable bias. Detailed information on SNPs are provided in Table S1.

#### 2.2.3 Mendelian Randomization Analysis

For MR analysis, the inverse variance weighted (IVW) method was used, supplemented by the weighted median (WM) and MR‐Egger methods for verification, ensuring the accuracy of the causal relationship between vitamin D levels and IDA (Bowden et al. 2017, 2016; Bowden, Davey Smith, and Burgess 2015). The Two Sample MR package in R software (version 4.3.1) was used to carry out the MR calculations.

#### 2.2.4 Heterogeneity Testing, Sensitivity Analysis, and Pleiotropy Analysis

The MR‐Egger and IVW methods were implemented for heterogeneity testing. There was considered to be heterogeneity among the SNPs if the *p*‐value was less than 0.05. The leave‐one‐out approach was used to carry out sensitivity analyses in order to examine the impact of specific SNPs on the causal link. The MR pleiotropy test function was used to carry out pleiotropy analyses. Pleiotropy was considered to be present when the *p*‐value was less than 0.05.

## 3 Results

### 3.1 Cross‐Sectional Study

#### 3.1.1 Basic Characteristic of Study Participants

The clinical features of the research population are shown in Table 1. Of the 3728 participants, 650 (17.4%) were anemic, and 3078 (82.6%) were non‐anemic. While diabetes, alcohol consumption history, marital status, renal insufficiency, RBC folate, and hypertension did not significantly differ between the two groups, significant differences were observed for all other clinical characteristics (*p* < 0.05).

**TABLE 1:** Basic characteristic of study participants.

Variables	Total (*n* = 3728)	Non‐anemic group (*n* = 3078)	Anemic group (*n* = 650)	Statistic	*p*
Age (year), M (Q₁, Q₃)	39.00 (30.00, 46.00)	38.00 (30.00, 47.00)	39.00 (31.00, 45.00)	*Z* = −1.26	0.208
PIR, M (Q₁, Q₃)	2.19 (1.18, 4.12)	2.43 (1.23, 4.37)	1.86 (0.96, 2.99)	*Z* = −7.48	< 0.001
BMI (kg/m^2^), M (Q₁, Q₃)	27.09 (23.19, 32.45)	26.66 (23.05, 32.13)	28.22 (23.94, 33.70)	*Z* = −3.75	< 0.001
Ferritin (μg/L), M (Q₁, Q₃)	51.50 (23.00, 98.62)	64.55 (36.00, 112.00)	8.32 (5.30, 13.28)	*Z* = −Inf	< 0.001
Hemoglobin (g/dL), M (Q₁, Q₃)	13.80 (13.20, 14.40)	14.00 (13.50, 14.60)	11.00 (10.10, 11.57)	*Z* = −Inf	< 0.001
MCV (fl), M (Q₁, Q₃)	88.80 (84.80, 91.90)	89.65 (86.90, 92.40)	78.10 (71.80, 83.70)	*Z* = −31.65	< 0.001
MCH (pg), M (Q₁, Q₃)	30.10 (28.40, 31.30)	30.50 (29.30, 31.50)	25.25 (22.90, 27.60)	*Z* = −33.27	< 0.001
Transferrin receptor (mg/L), M (Q₁, Q₃)	3.10 (2.50, 4.00)	2.89 (2.40, 3.47)	6.66 (4.60, 9.40)	*Z* = −33.83	< 0.001
Vitamin D (nmol/L), M (Q₁, Q₃)	59.85 (42.80, 78.10)	62.20 (46.90, 80.20)	43.05 (31.52, 61.60)	*Z* = −15.63	< 0.001
CRP (mg/L), M (Q₁, Q₃)	1.70 (0.70, 4.30)	1.62 (0.70, 4.13)	2.00 (0.70, 4.78)	*Z* = −2.61	0.009
SII (10^5^ cells/mL), M (Q₁, Q₃)	4.91 (3.54, 6.84)	4.82 (3.50, 6.63)	5.59 (3.74, 7.85)	*Z* = −5.28	< 0.001
Serum total folate (nmol/L), M (Q₁, Q₃)	35.60 (24.00, 43.80)	37.00 (25.10, 44.40)	30.20 (20.60, 41.98)	*Z* = −6.27	< 0.001
RBC folate (nmol/L), M (Q₁, Q₃)	1051.03 (764.75, 1230.00)	1051.03 (770.00, 1220.00)	1050.00 (734.17, 1300.00)	*Z* = −1.15	0.249
Total calcium (10 mmol/L), M (Q₁, Q₃)	23.25 (22.75, 23.75)	23.25 (22.75, 23.75)	22.75 (22.25, 23.50)	*Z* = −12.91	< 0.001
Gender, *n* (%)					
Male	617 (16.55)	592 (19.23)	25 (3.85)	*χ* ^2^ = 92.00	< 0.001
Female	3111 (83.45)	2486 (80.77)	625 (96.15)		
Race, *n* (%)					
Mexican American	589 (15.80)	470 (15.27)	119 (18.31)	*χ* ^2^ = 357.56	< 0.001
Other Hispanic	366 (9.82)	285 (9.26)	81 (12.46)		
Non‐Hispanic White	1589 (42.62)	1474 (47.89)	115 (17.69)		
Non‐Hispanic Black	707 (18.96)	432 (14.04)	275 (42.31)		
Other Race—Including Multi‐Racial	477 (12.80)	417 (13.55)	60 (9.23)		
Education level, *n* (%)					
Less than 9th grade	213 (5.71)	165 (5.36)	48 (7.38)	*χ* ^2^ = 42.83	< 0.001
9–11th grade	413 (11.08)	315 (10.23)	98 (15.08)		
High school graduate/GED or equivalent	788 (21.14)	627 (20.37)	161 (24.77)		
Some college or AA degree	1302 (34.92)	1078 (35.02)	224 (34.46)		
College graduate or above	1012 (27.15)	893 (29.01)	119 (18.31)		
Hypertension, *n* (%)					
Yes	816 (21.89)	670 (21.77)	146 (22.46)	*χ* ^2^ = 0.15	0.697
No	2912 (78.11)	2408 (78.23)	504 (77.54)		
Alcohol use, *n* (%)					
Yes	911 (24.44)	758 (24.62)	153 (23.54)	*χ* ^2^ = 0.344	0.558
No	2817 (75.56)	2320 (75.38)	497 (76.46)		
Diabetes, *n* (%)					
Yes	268 (7.19)	213 (6.92)	55 (8.46)	*χ* ^2^ = 1.911	0.167
No	3460 (92.81)	2865 (93.08)	595 (91.54)		
Renal insufficiency, *n* (%)					
Yes	80 (2.15)	64 (2.08)	16 (2.46)	*χ* ^2^ = 0.37	0.541
No	3648 (97.85)	3014 (97.92)	634 (97.54)		
Marital status, *n* (%)					
Married	1849 (49.61)	1551 (50.39)	298 (45.92)	*χ* ^2^ = 8.34	0.139
Widowed	97 (2.60)	82 (2.66)	15 (2.31)		
Divorced	386 (10.36)	322 (10.46)	64 (9.86)		
Separated	134 (3.60)	104 (3.38)	30 (4.62)		
Never married	854 (22.91)	685 (22.25)	169 (26.04)		
Living with partner	407 (10.92)	334 (10.85)	73 (11.25)		

Abbreviations: M, median; Q₁, 1st Quartile; Q₃, 3st Quartile; *Z*, Mann–Whitney test; *χ* ^2^, Chi‐square test.

#### 3.1.2 Logistic Regression Analysis

Factors with a *p*‐value less than 0.05 in the univariate analysis were incorporated into further univariate and multivariate logistic regression analyses. We found that high 25(OH)D levels (OR: 0.99, 95% CI: 0.98–0.99), possessing a college or AA degree (OR: 0.61, 95% CI: 0.41–0.91), being a college graduate or higher (OR: 0.56, 95% CI: 0.36–0.88), higher household income (OR: 0.93, 95% CI: 0.87–0.99), and being non‐Hispanic White (OR: 0.45, 95% CI: 0.33–0.61) were protective factors against IDA prevalence. In contrast, being non‐Hispanic Black, female and higher SII were identified as independent risk factors for IDA prevalence (all *p* < 0.05; Table 2).

**TABLE 2:** Results of logistic regression analysis of risk factors associated with IDA.

Variables	Univariate					Multivariate				
	*β*	*SE*	*Z*	*p*	OR (95% CI)	*Β*	*SE*	*Z*	*p*	OR (95% CI)
Gender										
Female					1.00 (Reference)					1.00 (Reference)
Male	1.78	0.21	8.54	< 0.001	0.17 (0.11–0.25)	−1.83	0.22	−8.42	< 0.001	0.16 (0.10–0.25)
Race										
Mexican					1.00 (Reference)					1.00 (Reference)
American										
Other Hispanic	0.12	0.16	0.71	0.477	1.12 (0.82–1.54)	0.30	0.17	1.74	0.082	1.34 (0.96–1.88)
Non‐Hispanic White	−1.18	0.14	−8.34	< 0.001	0.31 (0.23–0.41)	−0.81	0.16	−5.15	< 0.001	0.45 (0.33–0.61)
Non‐Hispanic Black	0.92	0.13	7.18	< 0.001	2.51 (1.95–3.23)	1.10	0.14	7.68	< 0.001	2.99 (2.26–3.96)
Other Race—Including Multi‐Racial	−0.57	0.17	−3.29	0.001	0.57 (0.41–0.80)	−0.19	0.19	−0.99	0.322	0.83 (0.58–1.20)
Educational level										
Less than 9th grade					1.00 (Reference)					1.00 (Reference)
9–11th grade	0.07	0.20	0.33	0.738	1.07 (0.72–1.58)	−0.25	0.22	−1.15	0.249	0.78 (0.50–1.20)
High school	−0.12	0.19	−0.67	0.503	0.88 (0.61–1.27)	−0.23	0.21	−1.11	0.269	0.80 (0.53–1.19)
Graduate/GED or equivalent	−0.34	0.18	−1.87	0.061	0.71 (0.50–1.02)	−0.50	0.20	−2.44	0.015	0.61 (0.41–0.91)
College graduate or above	−0.78	0.19	−4.09	< 0.001	0.46 (0.32–0.67)	−0.58	0.23	−2.54	0.011	0.56 (0.36–0.88)
Vitamin D (nmol/L)	−0.03	0.00	−13.62	< 0.001	0.97 (0.97–0.98)	−0.01	0.00	−5.29	< 0.001	0.99 (0.98–0.99)
CRP (mg/L)	0.02	0.01	2.89	0.004	1.02 (1.01–1.04)	−0.01	0.01	−0.60	0.548	0.99 (0.98–1.01)
PIR	−0.22	0.03	−7.81	< 0.001	0.80 (0.76–0.85)	−0.07	0.04	−2.12	0.034	0.93 (0.87–0.99)
BMI (kg/m^2^)	0.02	0.01	3.93	< 0.001	1.02 (1.01–1.03)	−0.01	0.01	−0.96	0.339	0.99 (0.98–1.01)
Serum total folate (nmol/L)	−0.02	0.00	−5.73	< 0.001	0.98 (0.98–0.99)	−0.00	0.00	−0.45	0.650	1.00 (0.99–1.00)
SII (10^5^ cells/mL)	0.07	0.01	5.97	< 0.001	1.07 (1.05–1.10)	0.09	0.01	6.49	< 0.001	1.09 (1.06–1.12)

Abbreviations: CI, confidence interval; OR, odds ratio.

#### 3.1.3 Correlation Between IDA and Serum Vitamin D Levels in All Subjects

As shown in Table 3, the initial correlation observed in the unadjusted model (OR: 0.97, 95% CI: 0.97–0.98, *p* < 0.001) was confirmed in Model 2 after adjusting for CRP and SII. Similarly, Model 3, which accounted for additional variables including gender, PIR, BMI, race, and education level, in addition to the variables included in Model 2, exhibited a similar trend. Further adjustments in Model 4 for RBC folate, age, marital status, hypertension, renal insufficiency, and serum total folate supported this correlation.

**TABLE 3:** Association between serum vitamin D level and IDA (all participants), NHANES 2005–2010, 2015–2018.

Variables	Model1		Model2		Model3		Model4	
	OR (95% CI)	*p*	OR (95% CI)	*p*	OR (95% CI)	*p*	OR (95% CI)	*p*
Vitamin D (nmol/L)	0.97 (0.97–0.98)	< 0.001	0.97 (0.97–0.98)	< 0.001	0.99 (0.98–0.99)	< 0.001	0.98 (0.98–0.99)	< 0.001

*Note:* Model1: Crude; Model2: Adjust: CRP, SII; Model3: Adjust: Model2+ gender, PIR, BMI, race, education level; Model4: Adjust: Model3+ RBC folate, age, marital status, hypertension, renal insufficiency, Serum total folate.

Abbreviations: CI, confidence interval; OR, odds ratio.

#### 3.1.4 Relationship Between IDA and Tertiles of Vitamin D

By quartiles we categorized the vitamin D levels into four groups so as to compare the relationship between various serum vitamin D levels and the incidence of IDA as shown in Table 4. Utilizing logistic regression analysis, we found that individuals within the T4 group (≥ 78.10 nmol/L) exhibited a reduce occurrence of IDA compared to those in the T1 group (≤ 42.80 nmol/L) across all four models. As initially observed in the crude model, which did not consider covariates, the T4 group displayed an 82% decrease in IDA risk. This protective pattern persisted in Models 2 through 4, which adjusted for various covariates.

**TABLE 4:** Association between serum vitamin D levels and IDA (Tertiles), NHANES 2005–2010, 2015–2018.

Variables	Model1		Model2		Model3		Model4	
	OR (95% CI)	*p*	OR (95% CI)	*p*	OR (95% CI)	*p*	OR (95% CI)	*p*
Vitamin D (nmol/L) quantile								
1	1.00 (Reference)		1.00 (Reference)		1.00 (Reference)		1.00 (Reference)	
2	0.36 (0.29–0.45)	< 0.001	0.36 (0.28–0.44)	< 0.001	0.54 (0.42–0.68)	< 0.001	0.49 (0.38–0.63)	< 0.001
3	0.23 (0.18–0.29)	< 0.001	0.23 (0.18–0.29)	< 0.001	0.43 (0.33–0.57)	< 0.001	0.36 (0.27–0.48)	< 0.001
4	0.18 (0.13–0.23)	< 0.001	0.18 (0.14–0.23)	< 0.001	0.43 (0.31–0.58)	< 0.001	0.33 (0.24–0.46)	< 0.001

*Note:* Model1: Crude; Model2: Adjust: CRP, SII; Model3: Adjust: Model2+ gender, PIR, BMI, race, education level; Model4: Adjust: Model3+ RBC folate, age, marital status, hypertension, renal insufficiency, Serum total folate.

Abbreviations: CI, confidence interval; OR, odds ratio.

#### 3.1.5 Risk Association of Serum Vitamin D and IDA in Women and Men

Table 5 illustrates the correlation between serum vitamin D levels and the risk of IDA in women. In the crude model, serum vitamin D exhibited a protective effect against IDA (OR: 0.97, 95% CI: 0.97–0.97, *p* < 0.001). However, as additional covariates were controlled for, this effect gradually diminished. Among the models, the weakest association was observed in Model 3 (OR: 0.99, 95% CI: 0.98–0.99, *p* < 0.001).

**TABLE 5:** Association of serum vitamin D levels and IDA in women.

Variables	Model1		Model2		Model3		Model4	
	OR (95% CI)	*p*	OR (95% CI)	*p*	OR (95% CI)	*p*	OR (95% CI)	*p*
Vitamin D (nmol/L)	0.97 (0.97–0.97)	< 0.001	0.97 (0.97–0.97)	< 0.001	0.99 (0.98–0.99)	< 0.001	0.98 (0.98–0.99)	< 0.001

*Note:* Model1: Crude; Model2: Adjust: CRP, SII; Model3: Adjust: Model2+ PIR, BMI, race, education level; Model4: Adjust: Model3+ RBC folate, age, marital status, hypertension, renal insufficiency, Serum total folate.

Abbreviations: CI, confidence interval; OR, odds ratio.

Table 6 shows the association between serum vitamin D levels and IDA in men. In the crude model, serum vitamin D showed no protective effect against IDA. Even after adjusting for covariates, no significant association between serum vitamin D levels and the risk of IDA was observed in men (Model 4: OR: 1.00, 95% CI: 0.98–1.02, *p* = 0.933).

**TABLE 6:** Association of serum vitamin D levels and IDA in men.

Variables	Model1		Model2		Model3		Model4	
	OR (95% CI)	*p*	OR (95% CI)	*p*	OR (95% CI)	*p*	OR (95% CI)	*p*
Vitamin D (nmol/L)	1.01 (1.00–1.02)	0.172	1.01 (0.99–1.02)	0.432	1.01 (0.99–1.03)	0.257	1.00 (0.98–1.02)	0.933

*Note:* Model1: Crude; Model2: Adjust: CRP, SII; Model3: Adjust: Model2+ PIR, BMI, race, education level; Model4: Adjust: Model3+ RBC folate, age, marital status, hypertension, renal insufficiency, Serum total folate.

Abbreviations: CI, confidence interval; OR, odds ratio.

#### 3.1.6 Modeling and Evaluation of the Nomogram

Based on the univariate and multivariate logistic regression analyses described above, a nomogram of the prevalence of patients with IDA was constructed using the following independent factors: gender, education level, race, serum vitamin D levels, PIR, SII (Figure S1). The calibration curves showed no significant deviations from the reference line, suggesting a high degree of predictive accuracy (Figure S2A). Furthermore, a more efficient application was produced in DCA when the model curves were at a threshold probability of 0.1–0.5, away from the extreme curves (Figure S2B).

#### 3.1.7 Modeling Using the Restricted Cubic Spline Method

**FIGURE 3:**
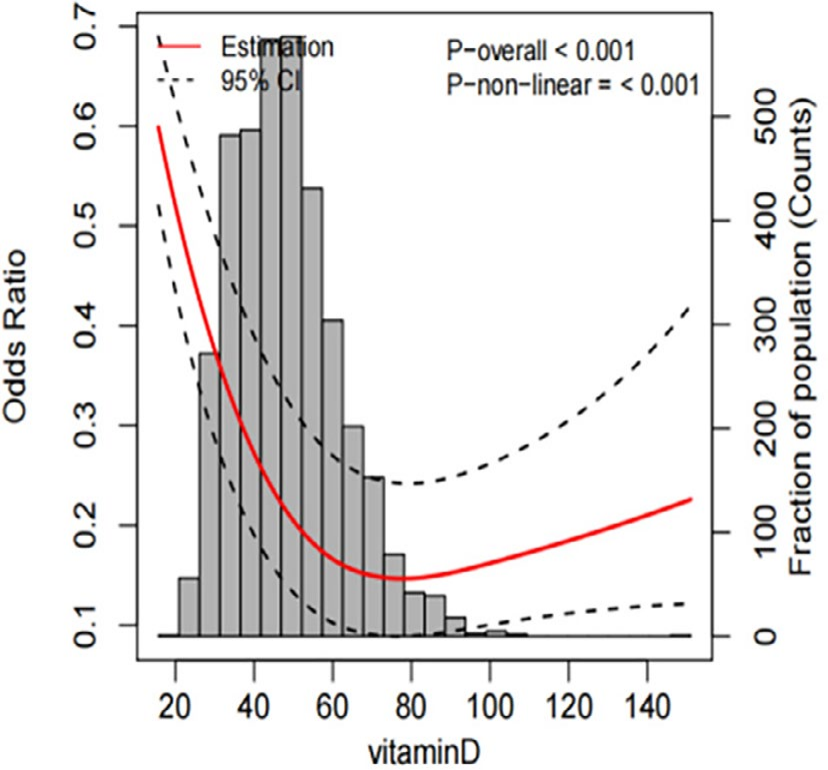
The restricted cubic spline for the associations between vitamin D levels and the risk of IDA.

As shown in Figure 3, a correlation between IDA and serum vitamin D levels was found. This correlation was modeled using the restricted cubic spline (RCS) method. The analysis revealed a declining prevalence of IDA with increasing vitamin D levels. Notably, a distinct “U”‐shaped relationship depicted in the plot was observed, indicating a significant decrease in IDA risk at higher serum vitamin D levels. The lowest risk of IDA was observed at approximately 65 nmol/L serum vitamin D levels, with a subsequent incremental trend (*p* for non‐linearity < 0.001), although the increase was relatively minor.

### 3.2 Mendelian Randomization Study

#### 3.2.1 Causal Relationship Between Serum Vitamin D Levels and IDA

**FIGURE 4:**
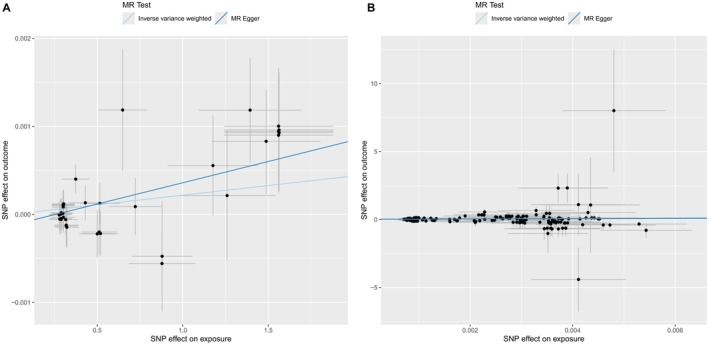
Scatter plots of genetic associations with serum vitamin D levels against the genetic associations with IDA. The blue line represents the inverse variance weighted estimate, the dark blue line represents the Mendelian randomization‐Egger estimate.

Causal relationship between genetically predicted serum vitamin D levels and reduced risk of IDA was found (IVW: β = 2.187E‐04, SE = 8.056E‐05, *p* = 0.007; WM: β = 3.110E‐04, SE = 1.146E‐04, *p* = 0.007; MR‐Egger: β = 4.829E‐04, SE = 1.672E‐04, *p* = 0.006; Table 7, Figure 4A). We performed a reverse MR analysis of vitamin D levels and IDA (MR‐Egger: β = 9.077, SE = 10.557, *p* = 0.391; WM: β = 22.617, SE = 9.015, *p* = 0.012; IVW: β = 20.816, SE = 5.909, *p* = 4.268E‐04; Table 8, Figure 4B). Therefore, our MR analysis suggested a potential causal relationship between serum vitamin D levels and IDA, with evidence supporting a mutually causative association between the two.

**TABLE 7:** MR estimates from each method of assessing the causal effect of serum vitamin D levels on the risk of IDA.

MR method	Number of SNPs	Beta	SE	Association *p*‐value	Cochran *Q* statistic	Heterogeneity *p*‐value
MR‐Egger	41	4.829E‐04	1.672E‐04	0.006	26.81	0.931
Weighted median	41	3.110E‐04	1.146E‐04	0.007	/	/
Inverse variance weighted	41	2.187E‐04	8.056E‐05	0.007	30.06	0.874

**TABLE 8:** MR estimates from each method of assessing the causal effect of IDA on vitamin D levels.

MR method	Number of SNPs	Beta	SE	Association *p*‐value	Cochran *Q* statistic	Heterogeneity *p*‐value
MR‐Egger	193	9.077	10.557	0.391	193.33	0.439
Weighted median	193	22.617	9.015	0.012	/	/
Inverse variance weighted	193	20.816	5.909	4.268E‐04	195.15	0.423

#### 3.2.2 Results of Heterogeneity and Sensitivity Analyses

In the MR analysis between serum vitamin D levels and IDA, no significant heterogeneity (*p* > 0.05; Table 7), or pleiotropy were detected (Egger intercept = −1.232e‐04, SE = 6.837e‐05, *p* = 0.079). Heterogeneity analysis (*p* > 0.05; Table 8) and horizontal multivariate analysis (Egger intercept = 0.021, SE = 0.016, *p* = 0.182) were also performed in the reverse MR analysis. Sensitivity analysis using the leave‐one‐out method did not identify any SNPs that significantly influenced the causal estimate (Figure S3). This indicated that the results of this study are stable; when any single SNP is removed, the combined causal effect of the remaining SNPs remains stable. In the funnel plot, calculated using both the IVW and MR‐Egger methods, the distribution of points on both sides was approximately symmetrical, indicating no unbalanced horizontal pleiotropy (Figure S4).

## 4 Discussion

In this study, we first conducted a cross‐sectional study using data from the NHANES 2005 to 2010 and 2015 to 2018 datasets, and then used summary data from GWAS to investigate the relationship between vitamin D levels and the risk of IDA. The observational data demonstrated a negative relationship between the risk of IDA and vitamin D levels, which was subsequently confirmed as causal through MR analysis.

Several studies have previously examined the interplay between vitamin D and IDA, primarily focusing on hepcidin, a key regulator of iron absorption. Hepcidin has shown an inverse correlation with iron absorption in both healthy females and those with iron deficiency (Aksan et al. 2019). For example, Bacchetta et al. investigated the impact of single and prolonged high‐dose vitamin D supplementation on hepcidin regulation. The study found that oral vitamin D2 increased the subjects' 25(OH)D levels from 68 nmol/L to 109 nmol/L. After 72 h, the concentration of hepcidin in the blood decreased by 34% (Bacchetta et al. 2014). Subsequent studies showed that 1 week after a single dose supplementation of 250,000 IU of vitamin D3, the subjects' hepcidin levels could decrease by 73% (Smith et al. 2017). In a cross‐sectional study involving 10,169 Korean women, including anemic (*n* = 1232), iron‐deficient (*n* = 2030), and IDA (*n* = 690) subsets of women, an elevated risk of IDA was associated with diminished vitamin D levels (Suh et al. 2016). Similarly, a study by Nikooyeh et al. investigated nearly a 1000 children aged 9–12 years and found a significant correlation between low vitamin D levels and an increased risk of anemia, regardless of age, gender, or BMI. Notably, vitamin D‐deficient children exhibited a three‐fold rise in anemia incidence compared to their vitamin D‐sufficient counterparts, with serum 25(OH)D levels < 44 nmol/L posing a heightened risk (Nikooyeh and Neyestani 2018). Moreover, a study involving 10,410 healthy children and adolescents in the United States also demonstrated a link between 25(OH)D deficiency and an increased risk of anemia. When 25(OH)D levels were below 12 ng/mL, Black children had lower hemoglobin levels (Atkinson et al. 2014). A placebo‐controlled, double‐blind randomized controlled trial reinforced these findings, demonstrating that daily supplementation with 38 μg (1500 IU) of vitamin D alongside an iron‐fortified breakfast cereal bolstered hemoglobin concentrations and erythrocyte volume in women with low iron reserves (Ahmad Fuzi and Mushtaq 2019). Furthermore, emerging evidence suggests an interaction between iron and vitamin D metabolism. Iron is believed to modulate the expression of vitamin D hydroxylase, as observed in some studies (El‐Adawy et al. 2019; Qiu et al. 2022). For example, a reduction in 25(OH)D levels was found in the serum of animals on an iron‐deficient diet compared to those on normal diets. This observation underscores the potential of iron supplementation to activate vitamin D hydroxylase, thereby elevating vitamin D levels (El‐Adawy et al. 2019). In our cross‐sectional study, we observed a heightened prevalence of IDA among patients with 25(OH)D deficiency. Similarly, we found an elevated prevalence of vitamin D deficiency among patients with IDA, consistent with findings from previous studies.

However, some studies indicate that vitamin D levels are not related to the occurrence of anemia (Alfhili et al. 2022; Soepnel et al. 2023; Braithwaite et al. 2019; Madar et al. 2016). A retrospective cross‐sectional study involving 14,229 people in Saudi Arabia showed that only adult males with anemia had significantly lower 25(OH)D3 levels, but no association between vitamin D levels and anemia was observed (Alfhili et al. 2022). Similarly, a cross‐sectional study of 520 South African women of childbearing age (18–25 years) indicated no significant correlation between vitamin D levels and anemia or iron deficiency (Soepnel et al. 2023). Braithwaite et al. conducted a randomized study on 195 women who delivered during spring (March to May), dividing them into an experimental group (*n* = 93) receiving vitamin D3 supplementation at 1000 IU/day and a control group (*n* = 102). The study analyzed hepcidin, ferritin, and inflammatory markers during early pregnancy (around 15 weeks) and late pregnancy (around 34 weeks) and found that vitamin D3 supplementation had no significant effect on these indicators (Braithwaite et al. 2019). Similarly, in a study on various ethnicities (South Asian, Middle Eastern, and African) residing in Norway, Madar et al. categorized participants with low vitamin D levels into three groups. Group 1 received 10 μg of vitamin D3 daily, group 2 received 25 μg of vitamin D3 daily for 16 weeks, and both were compared with a placebo group (Madar et al. 2016). The results indicated that vitamin D3 supplementation did not significantly affect hemoglobin levels or other parameters representing iron status.

The results of our study indicate that serum vitamin D levels are inversely correlated with the risk of IDA. Nevertheless, the overall odds ratio (OR) value is close to 1, indicating that the protective effect of vitamin D on IDA is relatively weak when considering unmeasured or uncontrolled confounding factors in traditional observational studies. Several studies have demonstrated an interactive relationship between nutrition and immunity (Roth‐Walter et al. 2024; Bacchetta et al. 2014; Peroni et al. 2023). In this study, patients with iron deficiency anemia were required to meet the criterion of serum ferritin < 30 μg/L. However, this criterion may have excluded some individuals with iron deficiency anemia accompanied by inflammation, which can lead to elevated ferritin levels.

MR analyses, which utilize large‐scale GWAS datasets, allow the lifetime effects of serum vitamin D levels to be estimated, rather than the effects of specific periods. This method offers important genetic insights into the connection between IDA risk and serum vitamin D levels. Our MR study data support the notion that serum vitamin D levels and IDA risk are causally related. However, in our study, the cross‐sectional and genetic data were sourced from different populations. Specifically, the MR analysis utilized individuals of European ancestry, whereas the cross‐sectional study involved a multi‐ethnic U.S. population. To eliminate potential confounding variables arising from population heterogeneity, future studies should be conducted on individuals of the same ethnicity.

The main advantage of our study is that our approach combines a two‐sample MR analysis with a large cross‐sectional research based on NHANES. Furthermore, MR studies can effectively address the issues of residual confounding, reverse causality, and measurement error that are frequently inherent in traditional epidemiological studies.

## Conflicts of Interest

The authors declare no conflicts of interest.

## Disclaimer

All claims expressed in this article are solely those of the authors and do not necessarily represent those of their affiliated organizations, or those of the publisher, the editors, and the reviewers. Any product that may be evaluated in this article, or claim that may be made by its manufacturer, is not guaranteed or endorsed by the publisher.

## Supporting information


**Figure S1.** Nomogram for predicting the prevalence of IDA.
**Figure S2**. Nomogram assessment of the prevalence of IDA.
**Figure S3**. Leave on out plot of the causal effects of single nucleotide polymorphisms associated with serum vitamin D levels on IDA.
**Figure S4**. Funnel plot to assess heterogeneity.

**Table S1.** Detailed information on SNPs between exposure factors (vitamin D) and outcome variables (IDA).

## Data Availability

The data that support the findings of this study are openly available inNational Health and Nutrition Examination Survey (NHANES) database at https://www.cdc.gov/nchs/nhanes/, IEU Open GWAS Project database at https://gwas.mrcieu.ac.uk/, and the Fenland study at https://www.finngen.fi/fi.
