# Supplementary material for: Serum Vitamin D Levels and Risk of Iron Deficiency Anemia in Adults: A Cross‐Sectional Study and Mendelian Randomization Analysis
Source: Food Sci Nutr. 2025 Feb 24;13(3):e4746. doi: 10.1002/fsn3.4746 (PMC11848641; doi:10.1002/fsn3.4746)
Supplement: Supplementary file 1 — Figure S1. Nomogram for predicting the prevalence of IDA. Figure S2. Nomogram assessment of the prevalence of IDA. Figure S3. Leave on out plot of the causal effects of single nucleotide polymorphisms associated with serum vitamin D levels on IDA. Figure S4. Funnel plot to assess heterogeneity. [file FSN3-13-e4746-s002.docx]

**Serum vitamin D levels and risk of iron deficiency anemia in adults: A cross-sectional study and Mendelian randomization analysis**

Leifei Chen ^1^, Nanyuan Gu ^1^, Kai Qiu ^1^ , Hui Chen ^1^, Fu Tian ^1^, Yang Chen ^1^and Longhuan Zeng ^1,*^

1 Department of High Dependency Unit，Hangzhou Geriatric Hospital; * Correspondence: [zenglonghuan@163.com](mailto:zenglonghuan@163.com)


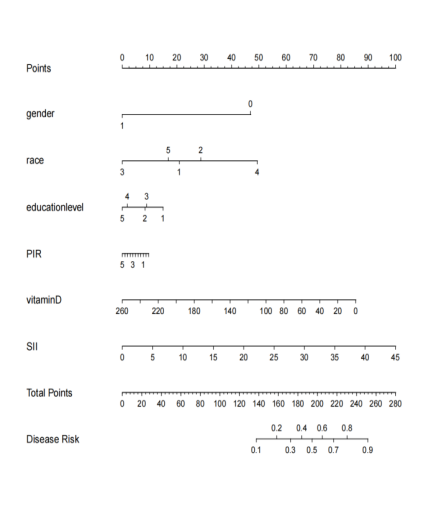


Supplementary Figure S1 Nomogram for predicting the prevalence of IDA.

Gender: 0. Female; 1. Male;

Race: 1. Mexican American; 2. Other Hispanic; 3. Non-Hispanic White; 4. Non-Hispanic Black; 5. Other Race - Including Multi-Racial

Education level: 1. Less than 9th grade; 2. 9-11th grade; 3. High school graduate/GED or equivalent; 4. Some college or AA degree; 5. College graduate or above

A
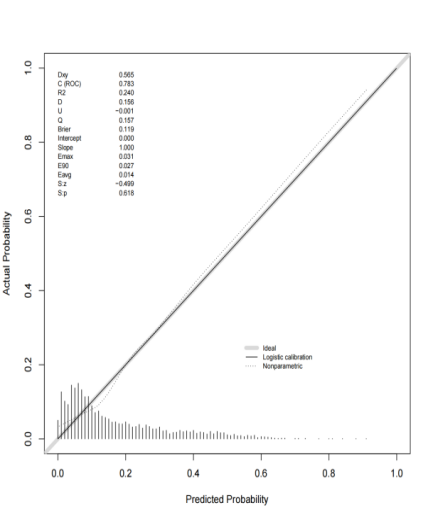
B
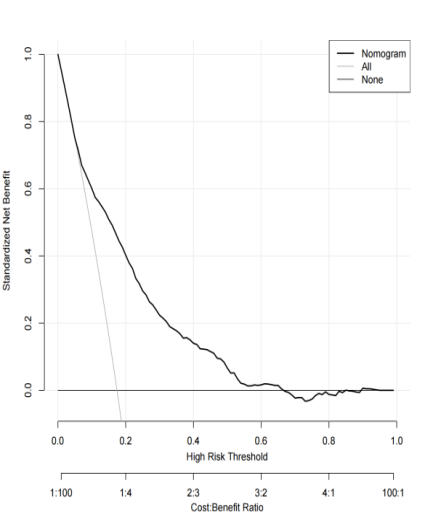


Supplementary Figure S2 Nomogram assessment of the prevalence of IDA

A.Calibration curve ; B. DCA. All. Extreme cases where all patients are sick ; None. Extreme cases where no patient is sick.

A
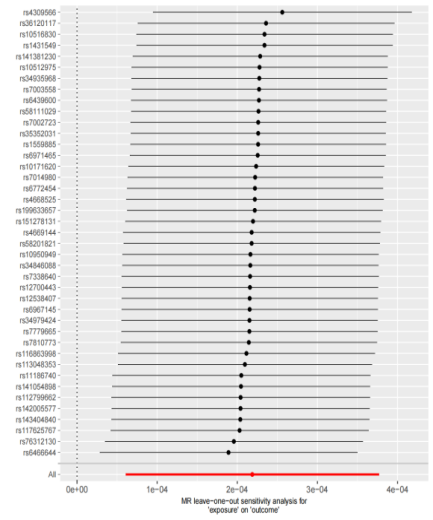
B
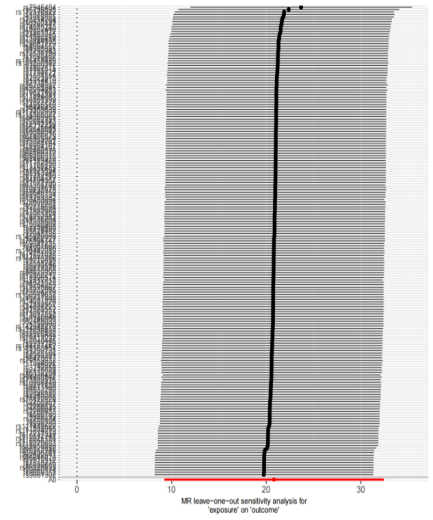


Supplementary Figure S3 leave on out plot of the causal effects of single nucleotide polymorphisms associated with serum vitamin D levels on IDA.

A
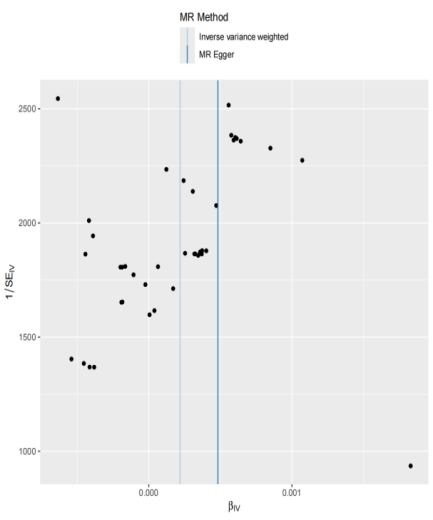
B
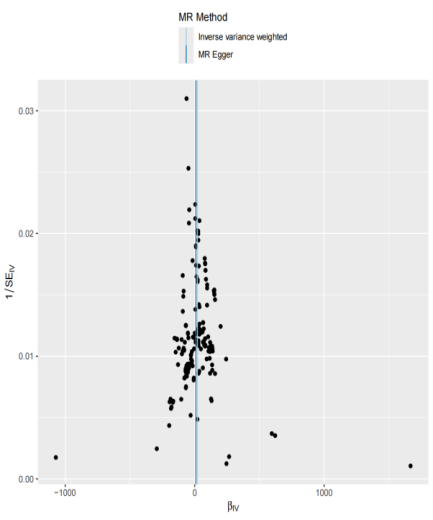


Supplementary Figure S4 Funnel plot to assess heterogeneity. The blue line represents the inverse‐variance weighted estimate, and the dark blue line represents the Mendelian randomization‐Egger estimate
